# Reply to Amundson: Time to go to work

**DOI:** 10.1073/pnas.2122842119

**Published:** 2022-03-18

**Authors:** Daniel L. Northrup, Bruno Basso, Michael Q. Wang, Cristine L. S. Morgan, Philip N. Benfey

**Affiliations:** ^a^Northrup.ag, St. Louis, MO 63118;; ^b^Department of Earth and Environmental Sciences, Michigan State University, East Lansing, MI 48823;; ^c^Systems Assessments, Argonne National Laboratory, Lemont, IL 60439;; ^d^Soil Health Institute, Morrisville, NC 27560;; ^e^HHMI, Duke University, Durham, NC 27708;; ^f^Biology Department, Duke University, Durham, NC 27708

Amundson’s letter (1) in response to our recent article (2) suggests that there are substantial challenges to the technologies that we envisioned being promising and/or needed to make the agricultural sector net negative. Aside from the difficulty in implementation, he makes no technical criticism of the findings. At the root of any disagreement between our article and these comments is the timeline over which change is necessary. Even if all other sectors reduce emissions to zero, the food sector will exceed the global carbon budget for 1.5 °C of warming in less than 40 y (3). Society cannot afford a slow approach to deep decarbonization.

Nonetheless, we do not claim that innovation or adoption will be easy, nor is this transition inevitable. Our goal was to introduce technologies (some already commercially available and adopted) and call for changes in policy and investment to broaden deployment and make the transition a reality. We do not propose that any of the transitional states should be the goal of the sector. As Amundson (1) notes, agriculture has never been in steady state. Indeed, the agricultural system must be reimagined to increase its productivity to feed a growing population while meeting sustainability targets, which represents a dramatic paradigm shift.

We chose a broad brush and a key metric—greenhouse gas emissions—for optimization of agricultural systems, as it fits current policy, incentives, and emerging markets. Amundson (1) highlights another interesting metric—elemental residence times in soils (4)—that could be a target for improving sustainability of agricultural systems. We are certain that using this metric will suggest additional technical thrusts for exploration.

To alter the aphorism from George Box, “all predictions are wrong, some are useful,” the purpose of our manuscript (2) was to articulate a technical vision that provides entry points for a paradigm shift. We have heard from colleagues in government, industry, nonprofit, and academia that the article is spurring discussion about policies and research programs to foster innovation and implementation. By this metric, we believe that it has been useful.

Amundson (1) notes that each innovation is a mini–Manhattan Project; the Manhattan Project succeeded in its goal in roughly 3 y. Mitigation of climate change is also likened to a moonshot, an effort that took 8 y. Given the consequences of failure, we hope that the climate challenge can be addressed on a decadal time scale. As Amundson concludes, we must do much work first. By laying out a plausible set of goals and identifying the areas for innovation, we aim to inspire that work.
